# Liposomal Lactoferrin Reduces Brain Neuroinflammation in Rats and Alleviates Jetlag and Improves Sleep Quality After Long-Haul Travel

**DOI:** 10.3390/neurosci6010019

**Published:** 2025-03-01

**Authors:** Shoko Uesaki, Masanori Yamato, Atsushi Ishikado, Yutaka Suekawa, Yasuhisa Tamura, Yosky Kataoka

**Affiliations:** 1 R&D Department, Sunstar Inc., 3-1 Asahimachi, Takatsuki 569-1195, Osaka, Japan; syoko.uesaki@jp.sunstar.com (S.U.); atsushi.ishikado@jp.sunstar.com (A.I.); yutaka.suekawa@jp.sunstar.com (Y.S.); 2 Laboratory for Cellular Function Imaging, RIKEN Center for Biosystems Dynamics Research, 6-7-3 Minatojima-minamimachi, Chuo-ku, Kobe 650-0047, Hyogo, Japan; yamatomasa@godzilla.kobe-u.ac.jp (M.Y.); tamurayasu@osaka-ohtani.ac.jp (Y.T.); 3 Graduate School of Science, Technology and Innovation, Kobe University, 6-7-3 Minatojima-minamimachi, Chuo-ku, Kobe 650-0047, Hyogo, Japan; 4 Laboratory of Clinical Pharmacology, Faculty of Pharmacy, Osaka Ohtani University, 3-11-1 Nishikori-Kita, Tondabayashi 584-8540, Osaka, Japan

**Keywords:** poor sleep, jetlag, neuroinflammation, hippocampus, poly I:C, liposomal lactoferrin

## Abstract

Insufficient sleep and circadian misalignment increase inflammatory agents. This triggers neuroinflammation and can result in health issues including depression, dementia, lifestyle-related diseases, and industrial accidents. Lactoferrin (LF) confers neuroprotective effects, which are derived from its anti-inflammatory, antioxidant, and iron metabolic properties; however, its roles in acute neuroinflammation and circadian rhythm disruption are yet to be elucidated. Therefore, we aimed to test the effects of LF on rat neuroinflammation and sleep and jetlag in humans. Rats received 7 days of an oral liposomal bovine LF (L-bLF) or vehicle followed by polyriboinosinic:polyribocytidylic acid (poly I:C) peritoneal injections (n = 5–6). Compared with the rats given poly I:C only, the rats given L-bLF and poly I:C had lower Il1b, Tnf, Casp1, Nfe212, Gclm, and Sod2 expression in the hippocampus. This open-label pilot study was carried out on tour conductors performing regular international tour responsibilities, and the data were compared between the initial tour without L-bLF intake and the subsequent tour with L-bLF intake. In the tour with L-bLF intake, L-bLF administration started from one week before the trip and was continued during the trip. In both periods, the tour conductors experienced limited sleep; however, both subjective and objective sleep quality was significantly better with the oral L-bLF intake than without. Overall, we found that prophylactic L-bLF supplementation reduced neuroinflammation in rat hippocampi and improved sleep quality and jetlag in tour conductors.

## 1. Introduction

Today’s 24 h society is associated with long work hours and shift work, which reduce sleep time and hinder rest quality. Insufficient sleep and circadian misalignment can lead to various health issues, such as depression, dementia, lifestyle-related diseases, and industrial accidents, among others. The extensive and substantial impact of these effects makes them a major public health concern.

Sleep disturbances and circadian disruption result in an overactive hypothalamic–pituitary–adrenal axis and sympathetic nervous system, which exacerbate neuroinflammatory conditions [1,2]. The enhanced neuroinflammation linked to sleep and circadian rhythm disruptions is implicated in the etiology of numerous diseases. As proinflammatory molecules involved in the sleep–circadian systems, interleukin (IL)-1 and tumor necrosis factor (TNF)-α contribute significantly to inducing and maintaining neuroinflammation [3,4,5]. Poor sleep symptoms, such as fatigue, cognitive impairment, depression, and sleepiness, are induced by exogenous IL-1 and TNF-α, and their inhibitors (IL-1 receptor antagonist and TNF-α soluble receptor) help alleviate these symptoms in people with insomnia [3]. Pharmaceutical and food ingredients like minocycline [6], curcumin [7], royal jelly [8], and caffeine [9] have gained attention in recent research for their ability to suppress elevations of IL-1 and TNF-α in the central nervous system (CNS); therefore, the emphasis on using food ingredients to prevent inflammation-related symptoms is growing.

Lactoferrin (LF) is an iron-binding glycoprotein that is part of the transferrin family. It has a molecular weight of approximately 80 kDa and is abundantly present in mammalian exocrine fluids, such as breast milk, tears, and saliva, as well as on the cell surfaces of neutrophils and gastrointestinal fluids [10,11]. Bovine lactoferrin (bLF) is manufactured as a raw material for functional foods and cosmetics. Human LF (hLF) and bLF have high sequence homology and share multiple functions, including iron homeostasis and anti-inflammatory, immunomodulatory, anti-oxidative, anti-tumor, anti-bacterial, and neuroprotective properties [11,12,13,14,15,16,17]. Furthermore, the recent SARS-CoV-2 epidemic brought renewed focus to the potential use of lactoferrin as a preventive and therapeutic agent, especially because of its cytokine-calming properties, with growing empirical evidence in its favor [11,12,18]. Several studies show that LF inhibits lipopolysaccharide (LPS)-induced proinflammatory cytokines, such as IL-1β, TNF-α, and IL-6, in peripheral tissues [19,20]. In addition, we previously demonstrated that bLF inhibits the activation of nuclear factor (NF)κB and mitogen-activated protein kinase pathways by binding to TNF-α receptor-associated factor 6, which strongly suppresses LPS-induced proinflammatory cytokine production in cultured cells [21].

LF production is observed in various human brain cells, including neurons, astrocytes, microglia, and oligodendrocytes, typically at low levels. However, LF expression is elevated with aging and in regions damaged by neurodegenerative diseases like Alzheimer’s and Parkinson’s disease (AD and PD) [11,22,23,24,25]. Several studies suggest that LF provides neuroprotection against CNS diseases through its anti-oxidative, iron homeostatic, and anti-inflammatory effects [11,22,23,24,25,26,27,28]. However, the efficacy of LF against acute and transient neuroinflammation, as seen in early infections and circadian misalignment, remains unclear.

LF administered orally is susceptible to being digested and has low intestinal membrane permeability; therefore, we developed bLF encapsulated within liposomes (liposomal bLF; L-bLF), which are spherical artificial vesicles composed of phosphatidylcholine bilayers [29,30,31,32]. We previously found that L-bLF given orally suppresses TNF-α production from mouse peripheral blood mononuclear cells (PBMCs) [30]; it also suppresses TNF-α expression in rat marginal periodontal tissue to a greater extent than non-liposomal bLF in LPS-induced experimental periodontitis [31]. We also demonstrated that oral administration of L-bLF significantly reduces LPS-induced proinflammatory cytokine production in PBMCs isolated from patients with periodontal disease [32]. Furthermore, participants with poor sleep had improved subjective sleep quality (feelings of deeper sleep, sleepiness upon waking, and recovery from fatigue) after consuming L-bLF orally [33]. However, the role of L-bLF in alleviating sleep disturbance through improvements in transient neuroinflammation remains uncertain.

Here, we first investigated whether oral administration of L-bLF suppressed hippocampal neuroinflammation in an animal model in which neuroinflammation was induced by intraperitoneal (i.p.) administration of polyriboinosinic:polyribocytidylic acid (poly I:C), a synthetic, double-stranded RNA. A single dose of poly I:C triggers a fever because of an acute innate immune response and a sustained (>1 week) adaptive response via toll-like receptor 3 (TLR3) [34,35,36]. The hippocampus is particularly susceptible to circadian disruption [37]; in addition, exaggerated proinflammatory responses, such as elevated hippocampal IL-1β, are observed in response to peripheral infection [38,39]. Therefore, we concentrated our study on the hippocampus.

Jetlag causes shortened sleep duration and circadian rhythm misalignment [40], and IL-1β and TNF-α play a role in regulating physiological sleep in the CNS [3,5]. Experimentally shortened sleep duration [5] and circadian misalignment [2] elevate levels of inflammatory mediators such as IL-1β and TNF-α, which causes neuroinflammation [41]. Viral infections like colds and influenza not only cause neuroinflammation but impair CNS function and disrupt sleep. Circadian misalignment can even facilitate a proinflammatory state under unchallenged conditions [42], while regulating the production of daytime cytokines to reduce their proinflammatory properties may alleviate sleepiness and fatigue linked to irregular sleep patterns [3,43,44]. Because of jetlag-induced disruptions to sleep and circadian rhythms during long-haul travel, we hypothesized that neuroinflammation would be induced in frequent travelers. Therefore, in the second part of the study, we tested the effectiveness of L-bLF supplementation on sleep and jetlag in tour conductors.

## 2. Materials and Methods

### 2.1. Experimental Design in Rats

Twenty-one male Sprague–Dawley rats (Japan SLC, Hamamatsu, Japan; 7 weeks old) were used. The animals were kept in the same way as in our previous study [34]. All experimental protocols were approved by the Ethics Committee on Animal Care and Use of the RIKEN Center for Life Science Technologies (Approval No.: MA2007-01-22, Approval date: 4 July 2019) and were performed in accordance with the Principles of Laboratory Animal Care (NIH publication No. 85–23, revised 1985). All efforts were made to minimize animal suffering and the number of animals used for this study.

### 2.2. Sample Preparation

L-bLF was composed of multi-lamellar vesicles and prepared by hydrating dietary soy phosphatidylcholine (Tsuji Oil Mill Co., Ltd., Tokyo, Japan) with an aqueous solution containing bLF (Morinaga Milk Industry Co., Ltd., Tokyo, Japan). As previously reported [32], 10.2% (*w*/*w*) soy phosphatidylcholine solubilized in glycerin and 19.8% (*w*/*w*) bLF were mixed at a ratio of 1.00:1.54 and emulsified (R&D Department, Sunstar Inc., Osaka, Japan). The emulsified solution was liposomalized using a high-pressure homogenizer (Star Burst Mini, Wet Pulverization and Dispersion Device, Sugino Machine Limited, Toyama, Japan). The average diameter of the liposomes, determined using a particle size analyzer, was approximately 60 nm. The control solution (glycerin) was prepared similarly.

### 2.3. Experimental Protocol

**Figure 1:**
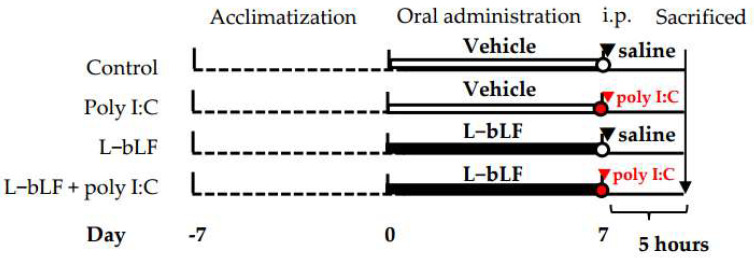
A timeline showing the experimental time points and treatments for the four groups of rats. i.p, intraperitoneal injection; poly I:C, polyriboinosinic:polyribocytidylic acid; L-bLF, liposomal bovine lactoferrin.

Figure 1 shows the experimental schedule. The rats were housed in the experimental room for at least one week prior to oral administration of L-bLF, and body weight and water intake were measured. The rats were randomly divided into four groups as follows:

- Control: Vehicle solution orally and i.p. administration of saline (n = 5);
- Poly I:C: Vehicle solution orally and i.p. administration of poly I:C (n = 5);
- L-bLF: L-bLF solution orally and i.p. administration of saline (n = 5);
- L-bLF + poly I:C: L-bLF solution orally and i.p. administration of poly I:C (n = 6).

L-bLF solution was administered at a dose of 500 mg/kg body weight/day through drinking water, from 7 days before the i.p. administration of poly I:C (GE Healthcare Life Science, Buckinghamshire, UK). Poly I:C is a synthetic, double-stranded RNA, which was dissolved in saline at a dose of 10 mg/kg body weight.

Five hours after the i.p. administration of poly I:C or saline, the rats were sacrificed under deep anesthesia and perfused with saline through the left ventricle. The hippocampus was extracted from the whole brains, immersed in RNAlater solution, and stored refrigerated for quantitative reverse transcription polymerase chain reaction (qRT-PCR) analysis.

### 2.4. qRT-PCR Analysis of Brain mRNA Expression in Rats

Total RNA was extracted from the hippocampus with the ISOGEN PB Kit (Nippon Gene, Tokyo, Japan), following the manufacturer’s instructions. Single-stranded cDNA was synthesized from 1 ug of total RNA using a reverse transcriptase with gDNA eraser (Takara Bio, Shiga, Japan). Complementary DNA (10 ng) was amplified by RT-PCR. qRT-PCR was carried out in the Thermal Cycler Dice Real Time System (Takara Bio, Shiga, Japan), using the standard protocols with KAPA SYBR qPCR Master MIX (Kapa Biosystems, Boston, MA, USA). All quantitative data were normalized to the expression level of *Rps18*. The primer sequences used are displayed in Table 1.

### 2.5. Participants and Study Design

The open-label pilot trial (UMIN-CTR, UMIN000054240) was performed in accordance with the Helsinki Declaration and was approved by the Ethics Committee of Sunstar Inc. (Approval No. 18-H1-05, Approval date: 6 July 2018, Osaka, Japan).

Seventeen tour conductors (1 male, 16 females; 41–57 years of age) who worked for JTB & JCB Human Solutions Corp. were included. The inclusion criteria were as follows: (1) no allergy to cow’s milk or soybeans; (2) no consumption of food containing 100 mg or more bLF within two weeks of participating in the study; (3) at least two overseas tours from Japan to areas with a time difference of 5 h or more; (4) experience of jetlag or travel fatigue; (5) able to follow the procedures of various surveys conducted during the test period (e.g., filling out various survey forms and wearing an Actigraph); and (6) no participation in other clinical studies at the start of the trial. Written informed consent prior to participation was obtained from all participants.

Each participant received 21 days’ worth of L-bLF and was instructed to take it for approximately one week prior to travel and during their trip (≤10 days). Six tablets (Kenkodojo Lactoferrin S; Sunstar Inc., Osaka, Japan) were given to each participant for each day (270 mg of L-bLF per day). The tablets were composed of a multi-lamellar vehicle, which was prepared by hydrating food-grade soy phosphatidylcholine (Tsuji Oil Mill Co., Ltd., Tokyo, Japan) in an aqueous solution containing bLF (Morinaga Milk Industry Co., Ltd., Tokyo, Japan). The participants were allowed to perform regular international tour responsibilities, and the data were compared between the initial period without L-bLF intake and the subsequent period with L-bLF intake.

### 2.6. Outcomes of the Open-Label Pilot Study in Humans

The primary outcome variable was the actigraphic data measured by the Actigraph wGT3X-BT (Acti Japan Co., Ltd., Chiba, Japan). All participants were asked to wear the Actigraph wGT3X-BT on their wrists and keep sleep diaries during their normal tour duties. Self-reported bedtime and wake time were used to supplement the actigraphic data for bedtimes and wake times. The data were assessed in 60 s epochs and analyzed using ActiLife 6 software (Acti Japan Co., Ltd., Chiba, Japan). Sleep–wake determination was performed using Sadeh et al.’s algorithm. Non-wear time was excluded from the analyses.

The PSQI [45] was administered at baseline to assess subjective sleep quality over the previous month. The PSQI has 9 items that generate 7 component scores for subjective sleep quality: sleep latency, sleep duration, habitual sleep efficiency, sleep disturbances, use of sleep medications, and daytime dysfunction. The global PSQI score was calculated by adding up the 7 component scores. The LJLQ [46] was used to assess the participants’ subjective ratings of jetlag on their first and second days after arriving overseas (i.e., after their first and second night’s sleep after traveling). The participants were also asked to fill out the OSA-MA [47], which has been standardized to assess sleep quality at night on the next morning. The OSA-MA consists of 16 items.

The LJLQ and OSA-MA scores were evaluated using visual analogue scales.

### 2.7. Statistical Analyses

Statistical analyses were performed using IBM SPSS Statistics version 29.0 (IBM Japan, Tokyo, Japan). The variables are represented as mean ± SEM. One-way ANOVAs with Tukey’s post hoc test for multiple comparisons was used for the qRT-PCR data. Paired *t*-tests were used to compare the actigraphic data and the PSQI, LJLQ, and OSA-MA values between the non-intake and L-bLF periods during overseas tour guiding. Significance was set at a *p* value less than 0.05.

## 3. Results

### 3.1. Animal Study Experimental Groups

Twenty-one Sprague–Dawley rats were divided into four groups: control (n = 5), poly I:C (n = 5), L-bLF (n = 5), and L-bLF + poly I:C (n = 6). In the L-bLF groups, L-bLF was administered to the rats for 7 days before an i.p. saline (control) or poly I:C injection. Water consumption and body weight measurements did not differ between the groups throughout the experiment. The mean water consumption during the experimental period was 28.8, 30.5, 28.8, and 28.6 g in the control, poly I:C, L-bLF, and L-bLF + poly I:C groups, respectively. The daily intake of L-bLF based on water consumption was estimated as 463.2 mg/kg for the L-bLF group and 472.3 mg/kg for the L-bLF + poly I:C group.

### 3.2. The Effects of L-bLF on Hippocampal mRNA Expression Levels in Poly I:C-Injected Rats

**Figure 2:**
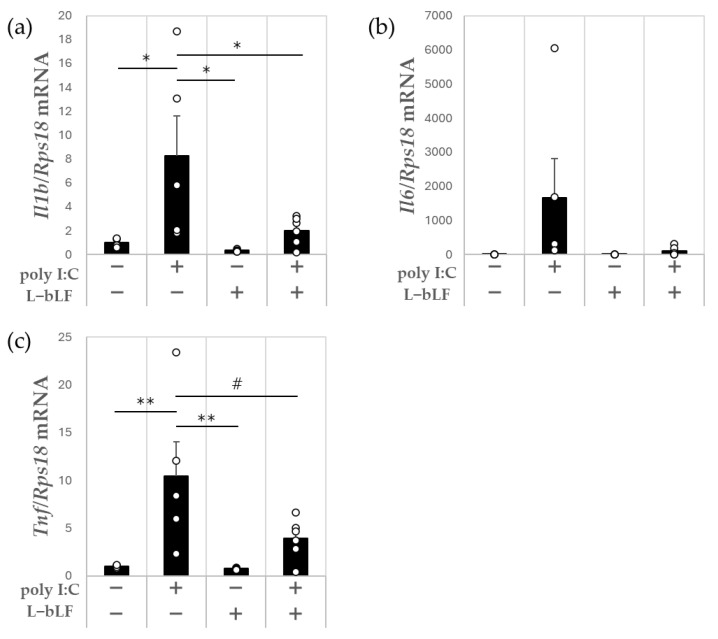
The effect of poly I:C and L-bLF on mRNA expression levels of inflammatory cytokines in the hippocampus. (**a**) *Il1b*, (**b**) *Il6*, (**c**) *Tnf*. Results are expressed relative to *Rps18*. Values are represented as mean ± SEM. A one-way ANOVA with Tukey’s post hoc test for multiple comparisons was used to compare the groups, # *p* < 0.10, * *p* < 0.05, ** *p* < 0.01, n = 5–6. Each open circle indicates the value in each animal. Poly I:C, polyriboinosinic:polyribocytidylic acid; L-bLF, liposomal bovine lactoferrin.

The *Il1b* and *Tnf* expression levels in the hippocampus were significantly higher, by 8.3- and 10.4-fold, respectively, in the poly I:C injection group compared with those in the control group; *Il6* expression was 1671.2-fold higher in the poly I:C group than in the control group, although high variation between the samples meant that this was not statistically significant (Figure 2a–c). Compared with those in the poly I:C group, the *Il1b* and *Tnf* expression levels were 76% (*p* < 0.05) and 63% lower (*p* < 0.1), respectively, in the L-bLF + poly I:C group; *Il6* expression was 93% lower, but this was not statistically significant (Figure 2a–c).

**Figure 3:**
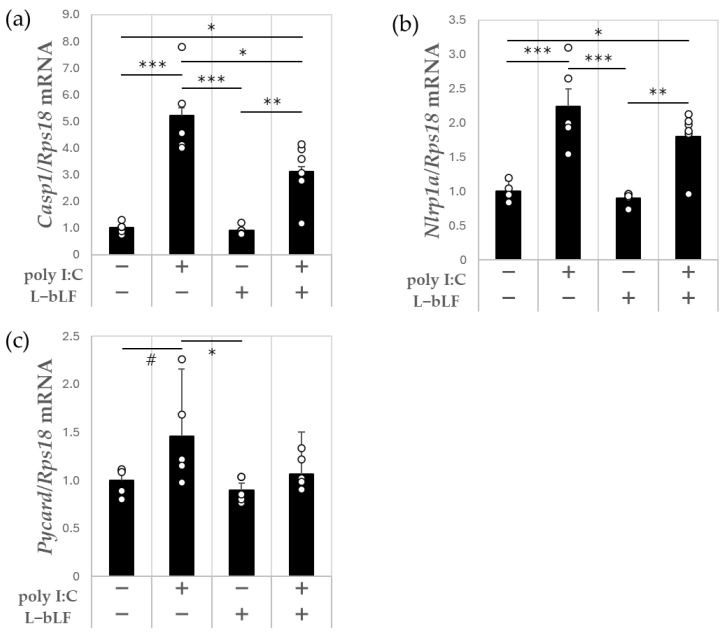
The effect of poly I:C and L-bLF on mRNA expression of the NLR1 inflammasome in the hippocampus. (**a**) *Casp1*, (**b**) *Nlrp1a*, (**c**) *Pycard*. Results are expressed relative to *Rps18*. Values are represented as mean ± SEM. A one-way ANOVA with Tukey’s post hoc test for multiple comparisons was used to compare the groups, # *p* < 0.10, * *p* < 0.05, ** *p* < 0.01, *** *p* < 0.001, n = 5–6. Each open circle indicates the value in each animal. Poly I:C, polyriboinosinic:polyribocytidylic acid; L-bLF, liposomal bovine lactoferrin; NLR1, NLR family pyrin domain containing 1.

After injection of poly I:C, the *Casp1* and *Nlrp1a* expression levels were significantly higher by 5.2- and 2.2-fold, respectively, while *Pycard* expression was 1.5-fold higher (*p* < 0.1) than in the control group (Figure 3a–c). Compared with the findings in the poly I:C group, *Casp1* expression was 40% lower in the L-bLF + poly I:C group (*p* < 0.05) (Figure 3a); *Nlrp1a* and *Pycard* expression levels were 20% and 27% lower, respectively, although these differences were not significant (Figure 3b,c).

**Figure 4:**
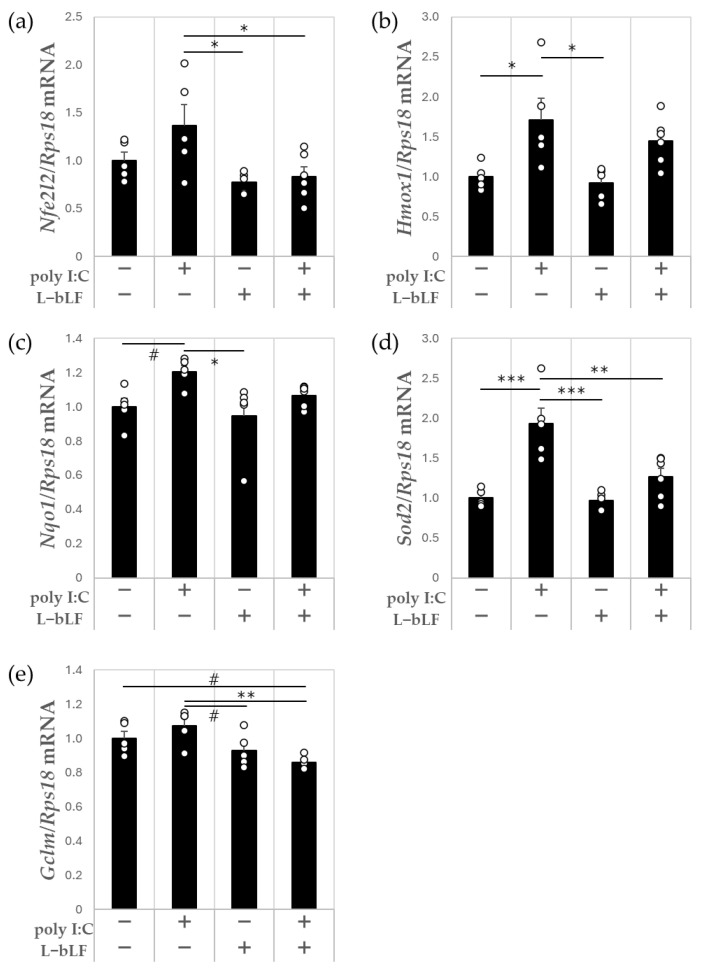
The effect of poly I:C and L-bLF on mRNA expression levels of Nrf2 pathway components in the hippocampus. (**a**) *Nfe2l2*, (**b**) *Hmox1*, (**c**) *Nqo1*, (**d**) *Sod2*, (**e**) *Gclm*. Results are expressed relative to *Rps18*. Values are represented as mean ± SEM. A one-way ANOVA with Tukey’s post hoc test for multiple comparisons was used to compare the groups, # *p* < 0.10, * *p* < 0.05, ** *p* < 0.01, *** *p* < 0.001, n = 5–6. Each open circle indicates the value in each animal. Poly I:C, polyriboinosinic:polyribocytidylic acid; L-bLF, liposomal bovine lactoferrin; Nrf2, nuclear factor erythroid 2-related factor 2.

After injection of poly I:C, the *Hmox1* and *Sod2* expression levels were significantly higher by 1.7- and 1.9-fold, respectively, compared with those in the control group (Figure 4b,d). *Nqo1* expression was 1.2-fold higher (*p* < 0.1), whereas the *Nfe2l2* and *Gclm* expression levels were non-significantly higher by 1.4- and 1.1-fold, respectively, in the poly I:C group compared with the findings in the control group (Figure 4a,c,e). Compared with the findings in the poly I:C group, the *Nfe2l2*, *Sod2*, and *Gclm* expression levels were significantly lower in the L-bLF + poly I:C group (by 39%, 34%, and 20%, respectively, all *p* < 0.05); the *Hmox1* and *Nqo1* expression levels were 15% and 12% lower in the L-bLF + poly I:C group, respectively, but this was not significant (Figure 4a–e).

### 3.3. Characteristics of the Study Participants

For the human study, tour conductors who regularly undertook long-haul travel and had experienced jetlag after travel were recruited. Sleep quality and jetlag were assessed after long-haul travel with or without L-bLF intake for 7 days before traveling. The participant characteristics are summarized in Table 2. Three participants were excluded for one of the following reasons: tour cancellation during the non-intake period, no actigraphic data during the control period, or a delay in test tablet intake during the L-bLF period. The final analysis included 14 participants. Most participants (92.9%) were female; they had an average age of 49 years (range 41–57 years), had an average of 22 years of overseas tour guiding experience (range, 10–34 years), and an average global Pittsburgh Sleep Quality Index (PSQI) score of 7.6 (range, 3–12). A global PSQI score of greater than 5.5 is considered a sign of sleep disturbance [45]. Therefore, in this study, all but one participant had sleep disturbances. Six participants took tours in the same direction during the non-intake and L-bLF periods. The remaining seven participants took tours in different directions during the non-intake and L-bLF periods.

### 3.4. The Effects of L-bLF Sleep Quality and Jetlag in Tour Conductors

The actigraphic assessment of changes is shown in Table 3. Sleep latency, total counts, sleep efficiency, total minutes in bed, wake time after sleep onset, number of awakenings, and average awakening length were all significantly lower with the L-bLF intake than without; all these differences indicate improved sleep with L-bLF. Total sleep time did not significantly change between conditions.

**Figure 5:**
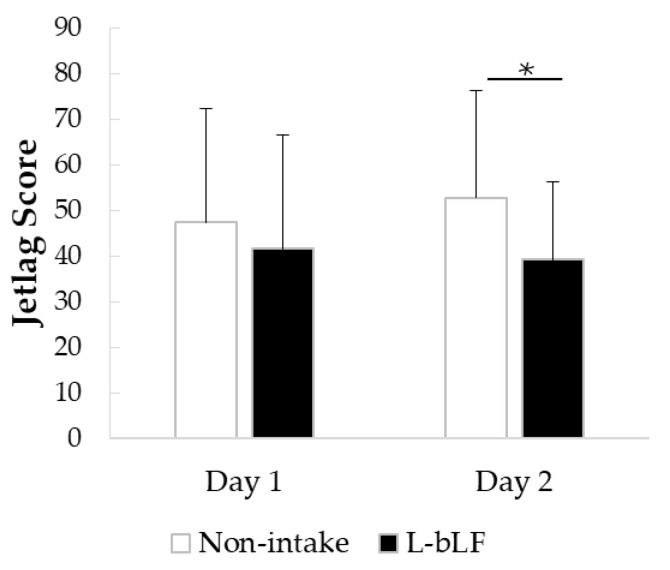
The effect of L-bLF supplementation on the jetlag score on the first and second day after travel was assessed with the LJLQ. Values are represented as mean ± SEM; significant differences were calculated using a paired *t*-test, n = 12. Because of missing data for two participants, data from 12 volunteers were included for assessment. * *p* < 0.05 versus the non-intake period. L-bLF, liposomal bovine lactoferrin; LJLQ, Liverpool Jetlag Questionnaire.

Two participants who did not complete the Liverpool Jetlag Questionnaire (LJLQ) [46] were excluded from the analysis; therefore, the jetlag analysis was conducted on 12 participants. Jetlag on the first day after travel did not differ significantly between the L-bLF period and the non-intake period (Figure 5, Table A1). However, jetlag on the second day after travel was significantly lower in the L-bLF period compared with that in the non-intake period.

Table 4 displays the subjective sleep quality scores assessed by a visual analogue scale based on the Oguri–Shirakawa–Azumi Sleep Inventory, Middle-Aged version (OSA-MA) [47]. In the “Sleepiness and fatigue on rising” section, “Not tired” tended to be higher in the L-bLF supplementation period than in the non-intake period (*p* = 0.09). In the “Initiation and maintenance of sleep” section, the following sleep quality markers were significantly higher with L-bLF supplementation than without: “Had a sound sleep”, “Had no trouble falling asleep”, and “Slept deeply”.

## 4. Discussion

In the present animal study, poly I:C was given intraperitoneally to provoke neuroinflammation through peripheral infection. Acute neuroinflammation was confirmed by upregulation of IL-1β mRNA (*Il1b*) (Figure 2a) and TNF-α mRNA (*Tnf*) (Figure 2c) in the hippocampus, as previously reported [34]. We also found that i.p. administration of poly I:C increased the expression levels of some genes that encode inflammasome-related molecules such as *Casp1*, *Nlrp1a* and *Pycard* and nuclear factor erythroid 2-related factor 2 (Nrf2)-regulated anti-oxidative enzymes such as *Sod2* and *Hmox-1* in the hippocampus. L-bLF pretreatment ameliorated polyI:C-induced increases in *Il1b* (Figure 2a), *Casp1* (Figure 3a), *Sod2* (Figure 4d), and *Gclm* expression (Figure 4e) in the hippocampus. These findings reveal a neuroprotective role for L-bLF in preventing polyI:C-induced damage to the hippocampus; this appears to occur via modulation of the inflammasome and Nrf2 antioxidant pathways. Our study is the first to focus on the possible activity of orally administered L-bLF against acute neuroinflammation in the hippocampus.

We noted a significant increase in proinflammatory cytokine mRNA expression (*Il1b* and *Tnf*) after poly I:C administration in the rats, an effect that was ameliorated by L-bLF supplementation. The TLR3 agonist poly I:C induces signal transduction to activate interferon regulatory factor 3 and NFκB via myeloid differentiation factor 88 (MyD88)-independent signaling pathways, which leads to the rapid regulation of type I interferons and proinflammatory cytokines [48]. Host defense responses, like inflammation and antioxidants, then get activated against microbial infection; by activating a common signaling pathway, TLR3 and TLR4 induce the production of proinflammatory cytokines, such as TNF-α, IL-6, and IL-1β [49]. We previously reported that intracellularly distributed bLF, mainly endocytosed through low-density lipoprotein receptor-related protein 1, interferes with Lys-63-linked polyubiquitination of TNF-α receptor-associated factor 6 and inhibits LPS-induced TLR4/NFκB signaling and mitogen-activated protein kinase activation [21]. In addition, orally administered bLF attenuates thioacetamide-induced increases in TLR4, MyD88, high mobility group box 1, NFκB, and TNF-α and decreases brain-derived neurotrophic factor contents in the brains of rats with hepatic encephalopathy [50]. Taken together, these results suggest that oral administration of L-bLF may inhibit increased transcription of IL-1β through activation of TLR-mediated NFκB-mediated intracellular signaling pathway upon i.p. administration of poly I:C.

In this animal study, L-bLF pretreatment suppressed the poly I:C-induced increase in caspase-1 mRNA (*Casp1*) in the hippocampus. The expression and activation of caspase-1 in neurons is crucial for the process of neuroinflammation [51]; indeed, chronic caspase-1 dysfunction can increase the risk of depression, PD, and AD [52]. Constant light-induced acute circadian disruption activates hippocampal caspase-1, rendering the brain vulnerable to subsequent inflammation and neurodegeneration [37]; L-bLF could hinder this process. In line with this, intragastrically administered LF lowers hippocampal caspase-1 levels and improves cognitive function in mice on a prolonged Western diet [53]. In our acute neuroinflammation model, L-bLF decreased hippocampal expression of *Casp1*, a key activator of IL-1β. If the change we observed in caspase-1 mRNA expression is also present at the protein level, it could, in turn, reduce the activity of IL-1β in the hippocampus, although further research is needed to confirm this.

Poly I:C administration intraperitoneally also led to an upregulation of Nrf2 gene expression (*Nfe2l2*) (Figure 4a). Nrf2 has an anti-inflammatory role, primarily because of its significant contribution to regulating reactive oxygen species and suppressing redox metabolism-induced inflammation [54]. Pharmacological upregulation of Nrf2 suppresses acute inflammatory responses in the brain [55]. Thus, enhancing Nrf2 expression could reduce the neuroinflammation triggered by poly I:C. Poly I:C stimulates TLR3 by stimulating inflammation and interferon-β expression, which induces reactive oxygen species production [56]. Both TLR-mediated innate immune responses and Nrf2-regulated antioxidant systems coordinate inflammation regulation [57]. The Nrf2 pathway cooperates with TLRs to prevent iNOS, cyclooxygenase-2, and proinflammatory cytokine production by inhibiting IκB phosphorylation and confining NFκB in the cytoplasm to prevent inflammation [48,58]. For instance, sulforaphane, an Nrf2 inducer, attenuates neuroinflammation by inhibiting TLR3-mediated NF-κB signaling and inducing Nrf2-mediated heme oxygenase-1 and NAD(P)H quinone oxidoreductase-1 expression [57]. Therefore, pretreatment with L-bLF appears to have minimized the neuroinflammation caused by TLR3 stimulation via intraperitoneal poly I:C administration, thus preserving the brain’s redox balance and preventing the need for an anti-oxidative response.

Higher levels of LF are found in lesion areas of multiple CNS diseases, including AD and PD, as well as in the aging brain [11,24,25,26]. Numerous studies show that LF can be transported into the brain via LF receptor-mediated transcytosis, and administered intranasally or intravenously, LF can cross the blood–brain barrier and blood–cerebrospinal fluid barriers to reach the brain of mice and rats [59,60,61]. However, whether orally administered L-bLF is transported into the brain to affect neuroinflammation in the hippocampus either directly or indirectly requires confirmation in future studies.

Using a rat model in our research, we found that L-bLF has the capability to inhibit experimentally caused neuroinflammation. It has been reported that the inflammatory mediators, specifically IL-1b and TNF-a, among others, influence sleep patterns and circadian rhythms [62], and their gene expression levels are heightened in response to sleep deprivation and circadian misalignment. Therefore, the second part of our study focused on the effect of L-bLF on sleep quality and jetlag in humans. In the present open-label pilot study, based on their global PSQI scores, all but one of the participants had sleep disturbances (Table 2). We found that approximately one week of L-bLF preventive administration before long-distance travel enhanced both objective (actigraphic assessment, Table 3) and subjective (OSA-MA, Table 4) sleep quality. Moreover, jetlag on the second day after travel was significantly improved after L-bLF intake (Figure 5). Compared with those values during the non-intake period, during the L-bLF intake period, total minutes in bed and total sleep time remained unchanged, indicating that supplementation of L-bLF enhanced sleep quality rather than sleep duration. Total sleep time during each period was remarkably short (288.70 and 286.67 min on average for the non-intake and L-bLF periods, respectively), suggesting that neuroinflammation may be a potential contributor to sleep disturbances caused by jetlag in tour conductors. As oral intake of L-bLF suppressed neuroinflammation in rats, our results suggest that L-bLF oral supplementation may alleviate the neuroinflammation caused by sleep deprivation and circadian misalignment in humans and lead to improved sleep quality, better regulation of circadian rhythm, and reduced jetlag symptoms on the second morning after travel.

Jetlag more commonly affects travelers going east than west [63]. During the periods of tours without L-bLF intake and those with L-bLF intake, the averaged time differences were 9.29 ± 0.82 h and 9.57 ± 0.84 h, respectively, with no significant difference noted between the two periods. In addition, during the non-intake and L-bLF periods, seven participants led tours in different directions. However, the frequency of eastward tours was roughly the same during the non-intake period and the L-bLF period, with four instances and three instances, respectively; therefore, the likely impact of tour direction on results is minimal (χ^2^ (2) = 1.143, *p* = 0.565).

LF, which acts as a multi-functional protein, exerts anti-stress and sedative effects and boosts light signal transmission to the suprachiasmatic nucleus [64,65]. The effects of bLF on these functions are mediated by nitric oxide (NO) production through NO synthase activation and/or opioid pathway stimulation. In addition, sleep disruptions and circadian misalignments can augment neuroinflammation, reactive oxygen species concentrations, and potentially gut microbiota dysbiosis [66]. We previously found that oral administration of L-bLF improved the feeling of deep sleep, fatigue, sleepiness on rising, and the enteric environment in poor sleepers [33], confirming the impact of L-bLF on the brain–gut axis.

The OSA-MA questionnaire results in our open-label pilot study indicated improvement in sleep initiation, maintenance, and refreshment following oral L-bLF administration. The regulation of the immune system, sleep, and circadian rhythms share common molecular mechanisms: IL-1β and TNF-α affect sleep and circadian rhythms to induce sleepiness, cognitive decline, depression, and fatigue, mimicking sleep loss symptoms [3,44]. A single circadian disruption event can exacerbate LPS-induced proinflammatory cytokine responses [3,63,67]. In addition, shift work disrupts the regular rhythm of PBMC secretion, which results in misaligned rhythms and an increase in proinflammatory cyto kines, including IL-1β, TNF-α, and IL-6 [59]. Thus, it is plausible that the anti-neuroinflammatory effects of L-bLF may have improved sleep quality (as indicated by actigraphy and psychological scales) and alleviated jetlag (as assessed by the LJLQ) caused by circadian disruption in our human trial.

This study had some limitations. We focused only on gene expression in rats; however, whether these changes resulted in alterations in protein expression and activity requires further investigation. Our results showed that L-bLF could improve sleep quality and jetlag in a preliminary, open-label, human trial. The intervention to obtain data from tour conductors was restricted to answering questionnaires and wearing Actigraphs, but it was not feasible to monitor inflammatory markers; thereby, additional research will be required in the future. Moreover, the participants’ knowledge that they were taking L-bLF could have affected the results, while the before-and-after design may have led to order effects. In addition, the study was executed while the tour conductors were on regular duties, precluding manipulation of travel directions and duration. Therefore, future double-blind randomized controlled trials are needed to confirm our results.

## 5. Conclusions

In summary, the present animal study demonstrated that L-bLF can be protective against neuroinflammation caused by poly I:C, which mimics viral infection. The neuroprotective effects were primarily mediated by its anti-inflammatory and anti-oxidative properties, as observed by the downregulation of inflammation and oxidative stress-related genes in the hippocampus. In addition, in an open-label pilot study, L-bLF supplementation improved sleep quality and jetlag symptoms caused by circadian rhythm dysregulation in tour conductors.

## Figures and Tables

**Table 1:** Primer sequences used in the qRT-PCR analysis.

Gene	Forward	Reverse
*Il1b*	CCCTGAACTCAACTGTGAAATAGCA	CCCAAGTCAAGGGCTTGGAA
*Il6*	ATTGTATGAACAGCGATGATGCAC	CCAGGTAGAAACGGAACTCCAGA
*Tnf*	TCAGTTCCATGGCCCAGAC	GTTGTCTTTGAGATCCATGCCATT
*Hmox1*	ATTTGTCCGAGGCCTTGAA	CCAGGGCCGTATAGATATGGTA
*Nfe2l2*	GATGATGCCAGCCAGCTGAA	GCGACTGACTAATGGCAGCAGA
*Nlrp1a*	GCCCTGGAGACAAAGAATCC	AGTGGGCATCGTCATGTGT
*Pycard*	TCTGTGCTTAGAGACATGGGCATAC	GCCATACAGAGCATCCAGCAA
*Casp1*	CTAGACTACAGATGCCAACCACTGA	GCATGATTCCCAACACAGGTACA
*Nqo1*	TGAGCCCGGATATTGTAGCTGA	GCATACGTGTAGGCGAATCCTG
*Sod2*	GGTGTGAGCTGCTCTTGATTGA	TTGATGGCCTTATGATGACAGTGA
*Gclm*	TGAATGGAGCTCCCAAATCAG	CATGGGACATGGTACATTCCAA
*Rp* *s18*	CTTCCACAGGAGGCCTACAC	GATGGTGATCACACGCTCCA

**Table 2:** Participant characteristics.

Age	Experience as an International Tour Guide (yrs)	Global PSQI Score	Non-Intake Period	L-bLF Period	Notes
43	20	7	Eastward	Eastward	Direction matched
50	20	7	Eastward	Eastward	Direction matched
50	28	7	Eastward	Eastward	Direction matched
53	20	-	Eastward	Eastward	Direction matched
54	10	8	Eastward	Eastward	Direction matched
57	34	12	Eastward	Eastward	Direction matched
57	30	7	Eastward	Eastward	Direction matched
41	15	9	Westward	Eastward	Direction mismatched
45	23	12	Eastward	Westward	Direction mismatched
46	21	3	Westward	Eastward	Direction mismatched
46	10	6	Eastward	Westward	Direction mismatched
49	25	6	Eastward	Westward	Direction mismatched
50	24	8	Westward	Eastward	Direction mismatched
53	20	-	Westward	Eastward	Direction mismatched
Average time difference (hours)			9.29 ± 0.82	9.57 ± 0.84	
Excluded participants					
43	17	10	Eastward	Eastward	Drop-out: delay in intake of test tablets during L-bLF period
53	20	-	Westward	Westward	Drop-out: missing actigraphic data during non-intake period
53	20	7	-	Westward	Drop-out: tour cancellation during non-intake period

PSQI, Pittsburgh Sleep Quality Index, L-bLF, liposomal bovine lactoferrin.

**Table 3:** Actigraphic assessment of changes in sleep with and without L-bLF administration.

		Mean ± SEM
Sleep latency (minutes)	Non-intake	5.98 ± 0.27
	L-bLF	4.20 ± 0.33 ***
Total counts	Non-intake	35,270.64 ± 4300.03
	L-bLF	20,998.01 ± 1863.39 ***
Sleep efficiency (%)	Non-intake	78.72 ± 2.83
	L-bLF	85.64 ± 2.33 ***
Total minutes in bed (minutes)	Non-intake	365.35 ± 17.51
	L-bLF	332.96 ± 13.25 #
Total sleep time (minutes)	Non-intake	288.70 ± 17.40
	L-bLF	286.67 ± 14.61
Wake after sleep onset (minutes)	Non-intake	70.59 ± 11.80
	L-bLF	42.02 ± 7.31 ***
Number of awakenings	Non-intake	17.49 ± 1.73
	L-bLF	13.51 ± 1.12 ***
Average awakening length (minutes)	Non-intake	4.30 ± 0.51
	L-bLF	3.12 ± 0.30 ***

Values are represented as mean ± SEM; significant differences were calculated using a paired *t*-test, # *p* < 0.10, *** *p* < 0.001, n = 14. L-bLF, liposomal bovine lactoferrin; NS, not significant.

**Table 4:** The effect of L-bLF on subjective sleep quality based on the OSA-MA.

	Non-Intake	L-bLF
	Mean ± SEM	Mean ± SEM
Sleepiness on rising		
Concentrated	53.13 ± 3.32	56.75 ± 4.31
Unstressed	48.07 ± 2.86	47.51 ± 2.80
Respond to the survey quickly	54.92 ± 3.68	57.09 ± 4.27
Quite awake	46.56 ± 3.06	50.15 ± 3.64
Initiation and maintenance of sleep		
Had a sound sleep	53.48 ± 5.66	65.40 ± 5.02 *
Went to sleep quickly	64.08 ± 5.04	71.51 ± 4.81
Had no trouble falling asleep	63.20 ± 4.90	72.88 ± 4.25 *
Had no arousal during sleep	51.01 ± 5.31	56.92 ± 4.85
Slept deeply	46.42 ± 4.50	56.82 ± 4.78 *
Frequent dreaming		
Had no nightmares	53.48 ± 5.66	65.40 ± 5.02
Had few dreams	64.08 ± 5.04	71.51 ± 4.81
Feeling of refreshment		
Not tired	33.10 ± 2.78	39.38 ± 2.49 #
Vigorousness	37.88 ± 2.99	38.85 ± 3.31
Pleasantly refreshed	44.69 ± 2.31	49.18 ± 2.64
Motivated to answer surveys	54.92 ± 3.68	57.09 ± 4.27
Sleep length		
Hungry on awakening	53.00 ± 3.80	52.48 ± 4.91
Had prolonged sleep	37.50 ± 4.42	39.11 ± 3.64

The OSA-MA consists of five sleep factors: I, Sleepiness on rising; II, Initiation and maintenance of sleep; III, Frequent dreaming; IV, Feeling of refreshment; and V, Sleep length. Values are represented as mean ± SEM, significant differences were calculated using a paired *t*-test, # *p* < 0.10, * *p* < 0.05, n = 14. L-bLF, liposomal bovine lactoferrin; OSA-MA, Oguri–Shirakawa–Azumi Sleep Inventory, Middle-Aged; NS, not significant.

## Data Availability

The datasets created and/or analyzed in this animal study can be obtained from the corresponding author upon reasonable request. The participant data in the human trials were safeguarded due to the requirement to uphold stringent privacy, with IRB-approved consent documents incorporating language to guarantee comprehensive protection measures. All relevant data are stored on file at Sunstar Inc.

## Appendix A

**Table A1:** Fatigue, sleep, mental performance and mood, meals, and bowel activity ratings according to the LJLQ during the non-intake period and the L-bLF period.

		Non-Intake	L-bLF
		Mean ± SEM	Mean ± SEM
Fatigue	Day 1	−0.38 ± 0.404	0.25 ± 0.734
	Day 2	−1.28 ± 0.571	−0.92 ± 0.437
Sleep	Day 1	0.39 ± 0.528	0.64 ± 0.695
	Day 2	0.70 ± 0.590	0.13 ± 0.601
Mental Performance and Mood	Day 1	−0.14 ± 0.328	0.05 ± 0.239
	Day 2	−0.36 ± 0.497	−0.29 ± 0.395
Meals	Day 1	−0.16 ± 0.280	0.22 ± 0.209
	Day 2	0.26 ± 0.246	−0.29 ± 0.387
Bowel Activity	Day 1	−0.88 ± 0.598	−0.02 ± 0.392
	Day 2	−0.54 ± 0.709	−0.29 ± 0.451

Answers were indicated by ticks placed on lines marked from −5 to 0 to +5, where −5 was defined as “much less than normal”, 0 was defined as “normal”, and +5 was defined as “much more than normal” for that time of day. There were no significant differences between groups for all items. LJLQ, Liverpool Jetlag Questionnaire.
